# A Low-Cost, Portable, and Wireless In-Shoe System Based on a Flexible Porous Graphene Pressure Sensor

**DOI:** 10.3390/ma14216475

**Published:** 2021-10-28

**Authors:** Tianrui Cui, Le Yang, Xiaolin Han, Jiandong Xu, Yi Yang, Tianling Ren

**Affiliations:** 1 School of Integrated Circuit, Tsinghua University, Beijing 100084, China; ctr19@mails.tsinghua.edu.cn (T.C.); yangl19@mails.tsinghua.edu.cn (L.Y.); hxl18@mails.tsinghua.edu.cn (X.H.); xjd18@mails.tsinghua.edu.cn (J.X.); 2 Beijing National Research Center for Information Science and Technology (BNRist), Tsinghua University, Beijing 100084, China

**Keywords:** pressure sensor, porous structure, gait analysis, in-shoe system, graphene

## Abstract

Monitoring gait patterns in daily life will provide a lot of biological information related to human health. At present, common gait pressure analysis systems, such as pressure platforms and in-shoe systems, adopt rigid sensors and are wired and uncomfortable. In this paper, a biomimetic porous graphene–SBR (styrene-butadiene rubber) pressure sensor (PGSPS) with high flexibility, sensitivity (1.05 kPa^−1^), and a wide measuring range (0–150 kPa) is designed and integrated into an insole system to collect, process, transmit, and display plantar pressure data for gait analysis in real-time via a smartphone. The system consists of 16 PGSPSs that were used to analyze different gait signals, including walking, running, and jumping, to verify its daily application range. After comparing the test results with a high-precision digital multimeter, the system is proven to be more portable and suitable for daily use, and the accuracy of the waveform meets the judgment requirements. The system can play an important role in monitoring the safety of the elderly, which is very helpful in today’s society with an increasingly aging population. Furthermore, an intelligent gait diagnosis algorithm can be added to realize a smart gait monitoring system.

## 1. Introduction

Gait is the behavioral characteristic of human walking, which can reflect people’s problems in physiology, athletic ability, and psychology [1]. In clinical practice, biomechanics and kinematics can reveal key links and influencing factors of gait abnormality to assist rehabilitation evaluation and treatment [2,3]. Therefore, gait analysis is an important assessment tool for daily life guidance and rehabilitation evaluation. For example, rehabilitation medicine can quickly judge the degree of risk of sports injuries according to the load conditions on the sole [4]. In addition, gait analysis plays an important role in monitoring the safety of the elderly [5], which is helpful in today’s society where population aging is increasing. Currently, gait recognition systems primarily have the following application scenarios: clinical gait analysis [6,7], sports shoe development [8,9,10], biometrics [11,12,13], orthopedic disease diagnosis [14,15], rehabilitation medicine [16,17,18], and so on [19,20]. Therefore, gait analysis has drawn tremendous attention from researchers.

The traditional gait recognition system is mainly carpet-type [21,22,23]. It focuses on the capture and judgment of postures and has limited applications, only in specific areas and locations, and cannot achieve continuous monitoring. To identify gait patterns, many researchers have attempted different methods. Anna et al. designed an inertial system to quantify gait symmetry and gait normality [24]. Tay et al. developed a wearable wireless gait monitoring system to assist the rehabilitation of patients with Parkinson’s disease [25]. The prototype system uses on-body acceleration sensors to measure patients’ movements. Dominguez et al. proposed a digital goniometer based on an encoder to achieve knee joint measurements [26]. Bamberg et al. used a gait analysis system with integrated wireless sensors for gait pattern recognition [27]. Pressure sensors are also used in gait analysis. Bae et al. proposed a mobile gait monitoring system, which consists of shoes, a data acquisition board, and a mobile display [28]. Chen et al. designed a shoe-integrated system that installed four force-sensing resistors and one bend sensor on the insole to capture force and flexion information, thereby enabling abnormal gait monitoring [29].

Compared to sensors applied to other parts of the body [30,31], wearable pressure sensors used for gait monitoring not only need to withstand extreme pressure (ground reaction force often exceeds human gravity), but still need to maintain high-sensitivity measurement for accurate gait analysis; besides, they should be flexible. These requirements put forward a high demand on the material and structure of the sensor. A solution is found in bionics. For vertebrates, bones are the hard organs that make up the endoskeleton, which supports and protect the body. The soft skeletal muscles attached to bones undertake functions such as systemic circulation and physiological information transmission, and their softness ensures adaptive deformation with changes in body shape. The structure of the sensor is similar to vertebrates’ bodies, consisting of an SBR foam skeleton and a graphene functional layer. As with the porous surface of the bone, which is suitable for skeletal muscles’ attachment and growth [32], the porous SBR foam provides plenty of attachment points for graphene. Its high resilience and flexibility ensure a stable structure of the sensor under extreme pressure with good recovery characteristics, in addition to making it comfortable to wear. The graphene layer attaches to it and forms a dense, three-dimensional conductive network, which can sensitively reflect changes in gait. Moreover, flexibility and air permeability, which are derived from the porous structure, are integrated into the sensor [33,34,35].

In this paper, we realized a low-cost, portable, and wireless in-shoe real-time gait monitoring system based on a biomimetic flexible porous graphene–SBR (styrene-butadiene rubber) pressure sensor (PGSPS). Compared with the sensors mentioned above [30,31], the PGSPS has better performance in terms of sensitivity, response time, linearity, and stability, which is very suitable for gait recognition. After testing its mechanical properties, sixteen PGSPSs were placed at specific positions of an insole system based on the distribution of human plantar pressure when walking. Subsequently, after verifying its ability to monitor gait signals with a high-precision digital multimeter, and comparing its performance between in-shoe and out-shoe usage, the signal collection insole, acquisition circuit, and gait recognition algorithm were combined to form a flexible real-time gait monitoring system. The system can accurately identify gaits such as normal gait, toe-in, toe-out, lame feet, and heel feet, and can be used to detect athletic states such as running and jumping. The insole system has the potential to detect diseases and can be used for the daily tracking of motion and rehabilitation training, which shows great potential in the daily health and medical field.

## 2. Materials and Methods

### 2.1. Fabrication of the Porous Graphene–SBR Pressure Sensor

**Figure 1:**
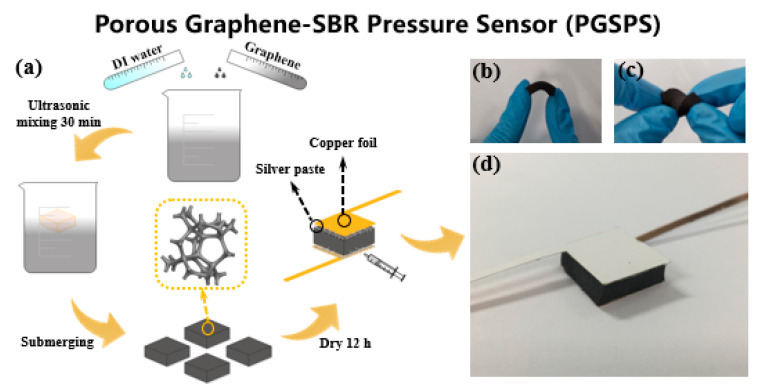
(**a**) Fabrication process of the PGSPS. (**b**,**c**) Photographs of folded and twisted PGSPS. (**d**) Photograph of PGSPS.

Figure 1a depicts the fabrication process of the PGSPS. Graphene nanoplate paste (0.5 wt%, 1–5 μm flake diameter, XFNANO) was mixed with deionized water at a volume ratio of 1:5 and placed in an ultrasonic cleaning machine (Shenhuatai Ultrasonic Cleaning Equipment Co., LTD, Shenzhen, China) for 30 min to make the graphene nanoplates disperse evenly. As a general rubber, styrene-butadiene rubber (SBR) is widely used and its foam has beneficial properties, including light weight, excellent insulation, and high flexibility. The SBR foam (Table 1) was manufactured through two-stage vulcanization, foaming styrene-butadiene rubber at a factory (Dongguan Juntai Foam Products Co., LTD, Dongguan, China) according to our requirements, and was tailored to a specific size (1.5 cm × 1 cm), which was limited by the scale of the sole and the accuracy (a smaller sensor results in insufficient sensitivity). Through submersion into the graphene suspension, the porous SBR foam was wrapped with graphene nanoplates. It was then dried naturally at room temperature for 24 h. Finally, the electrodes were welded with silver paste to both ends of the dried PGSPS for testing (Figure 1d). It is worth mentioning that proper mixing of deionized water and graphene can facilitate the uniform and adequate distribution of graphene nanoplates on the SBR foam without reducing its elasticity. As shown in Figure 1b,c, the prepared PGSPS exhibits good flexibility and is easy to bend, stretch, and fold, indicating that it has great application potential in the wearable electronics field.

To achieve low-cost preparation of the PGSPS and realize sustainable manufacturing, the main materials, including the graphene suspension, SBR foam, and silver paste, used in the fabrication process are all achieved by utilizing standardized production, ensuring that the cost is low and stable. In the process of the experiment, since excessive graphene dispersion was collected during the preparation of PGSPS, we improved material utilization through redissolution and ultrasonic dispersion, reducing the average consumption of graphene dispersion per PGSPS to 0.9 mL. In the preparation process, apart from the ultrasonic treatment to further reduce the graphene sheet diameter (in order to achieve uniform dispersion), there is no need for other high-energy-consumption and high-cost processes. The low-cost and low-energy-consumption preparation of PGSPS has been preliminary realized in the laboratory, which provides the possibility for subsequent large-scale manufacturing.

Furthermore, in the application of gait monitoring, multiple PGSPSs with the same performance are used at the same time, which requires performance stability and uniformity of the PGSPS. We found solutions in the preparation process: Firstly, the graphene sheet diameter was reduced by ultrasound to facilitate its uniform adhesion to the porous structure of the SBR foam. Second, for SBR foams that have been cut to a uniform size, they need to be repeatedly extruded after being immersed in the graphene suspension to ensure that the graphene is fully absorbed into the foam. Finally, the not-tightly-attached graphene should be flushed with water.

### 2.2. Design of the Smart Sole System

**Figure 2:**
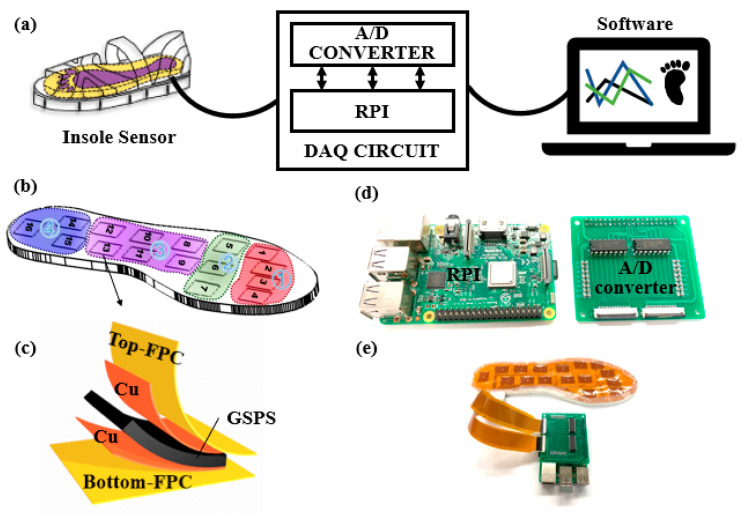
The smart sole system. (**a**) Measurement system. (**b**) Four regions of the insole-shaped FPC. Region one includes the hallux (1), 2nd toe (2), 3rd toe (3), and the 4th and 5th toes (combined) (4). Region two include the 1st, 3rd, and 5th metatarsus. Region three (8~13) and four (14~16) represent the midfoot and heel, respectively. (**c**) Structure of the insole sensor. (**d**,**e**) Photographs of the RPI, A/D converter, and DAQ system.

A miniaturized system was designed to realize long-term real-time monitoring of foot pressure during walking. Figure 2a illustrates that the smart sole system consists of three parts: a PGSPS-based sensor system with a double-faced insole-shaped flexible printed circuit board (FPC), a raspberry PI (RPI) system, a data acquisition (DAQ) circuit, and a data processing and displaying software.

As shown in Figure 2b, sixteen PGSPSs are placed in specific areas of the sole, which are divided into different regions according to the structure of the human foot and the distribution of foot pressure [36]. These regions are the phalanx (region one), metatarsus (region two), midfoot (region three), and heel (region four); each includes multiple sensors for accurate pressure measurements. Figure 2c shows the structure of a sensing point that consists of a PGSPS and two FPCs. The two FPCs act as the faces of the insole to encapsulate the PGSPS system and realize connectivity. Each FPC contains exposed conductive film at the sixteen positions to realize the electrical connection with a PGSPS. Each PGSPS leads to the detection interface through the FPC and connects the data processing module for testing. By applying a reference voltage to the PGSPSs, the MCP3008 A/D converter can obtain the analog voltage (V_A_) by calculating the resistance change in a PGSPS by the formula V_A_ = R_ref_/(R_ref_ + R_sensor_) V_CC_. Then, the MCP3008 changes the V_A_ to a digital signal (V_D_) by the formula V_D_ = (2^10^ V_A_)/V_ref._ After that, the signal processing module (as shown in Figure 2d) processes the signals and transmits them through Bluetooth to the smartphone software to display gait information. During real-time monitoring, the system conducts 150 reads per second. The whole smart sole system is shown in Figure 2e (without the smartphone for signal display). This method is suitable for the low-cost mass manufacturing of intelligent insoles, and it is easy to achieve customized designs for people with different foot types. It can be directly used as the actual insole in shoes or sandals for daily health and exercise monitoring. In addition, for a single PGSPS, by recording the curve of current varying with pressure at a constant voltage, we can obtain the power consumption. In the full-scale test of 0–150 kPa, with 80 mm/min extrusion speed, the average power of the PGSPS is 0.21 mW. This low power consumption means that the PGSPS can be used in real-time long-term monitoring.

### 2.3. Characterization and Testing Methods

The micromorphology and structure of the PGSPS were characterized by a field emission electron scanning microscope (FESEM) (JSM-7001F) (JEOL, Tokyo, Japan). Raman characterization was performed using LabRAM HR Evolution (HORIBA, Kyoto, Japan). The mechanical properties of the PGSPS were observed and recorded in real-time with a mechanical tester (SHIMADZU AGS-X/EZ-X) (SHIMADZU, Kyoto, Japan) and a digital multimeter (RIGOLDM3068) (RIGOL Technologies, Beijing, China). The preliminary gait test that was performed using the insole consisted of sixteen PGSPSs with the help of a DMM7510 7^1/2^ DIGIT MULTIMETER (Tektronix, Johnston, OH, USA). In the final in-shoe real-time monitoring system based on the PGSPS, the gait signals of an adult were monitored real-time, gait information was transmitted wirelessly and displayed on a smartphone.

## 3. Results and Discussion

### 3.1. Characterization of the PGSPS

Nature has always inspired designs, especially bionic structures for wearable applications. For vertebrates, bones are the hard organs that make up the endoskeleton, which supports and protects the body. The soft organs and tissues attached to bones undertake functions such as systemic circulation and physiological information transmission. Their softness ensures that they change with body shape and follow the skeleton’s movement. The design idea of the PGSPS is similar to vertebrates’ bodies, consisting of an SBR foam skeleton and a graphene functional layer. The porous structure of SBR foam not only provides plenty of attachment points for graphene but also supports the PGSPS. The graphene layer that attaches to it forms a dense, three-dimensional, and flexible conductive network, which is suitable for electrical signal transmission under deformation conditions. Besides, the magnificent flexibility of graphene [37] ensures that it closely follows the deformation caused by the movement of people and the SBR skeleton, which facilitates the fast response of the sensor. It is worth noting that the proper SBR foam parameters are critical for large-range pressure-sensing for insole applications, and proper mixing of deionized water and graphene facilitates the uniform and adequate distribution of graphene nanoplates on the SBR foam without reducing its elasticity. Therefore, the SBR foam was customized to meet the requirements of pore size and flexibility, which are suitable for the flexible, high-range pressure application.

**Figure 3:**
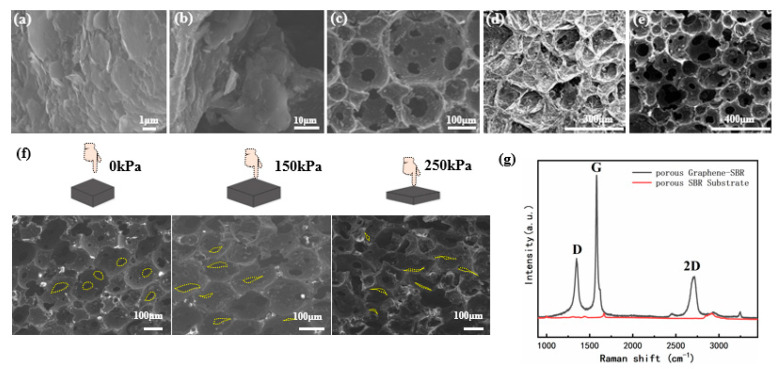
(**a**–**e**) SEM image of the PGSPS: the magnification increases gradually from (**a**) to (**e**). (**f**) Photographs of squeezed PGSPS, under 0 kPa, 150 kPa, and 250 kPa of pressure, respectively. (**g**) Raman spectra of graphene–SBR and SBR.

Figure 3a–e shows the morphology of the PGSPS under different magnifications. The three-dimensional porous structure can be observed by FESEM. The SBR foam consists of porous microspheres with hole sizes of 20–250 μm. The graphene sheet diameters are 1–5 μm; they cover the SBR skeleton uniformly to provide a conductive network. It can also be seen from the enlarged SEM images that several layers of graphene flakes are attached to the skeleton structure with good contact, which helps the sensor resistance to change stably with pressure. Figure 3f shows the pore diameter change under different pressures, corresponding to 0 kPa (without pressure), 150 kPa (maximum measuring range for insole application), and 250 kPa (maximum measuring range of the sensor), respectively. The graphene conductive networks become denser under compression, which results in a decrease in resistance as the pressure increases. As depicted in Figure 3g, obvious characteristic peaks were observed at ~1350 cm^−1^, ~1580 cm^−1^, and ~2700 cm^−1^ in the Raman spectrum, corresponding to the D, G, and 2D characteristic peaks of graphene, respectively. I_2D_/I_G_ < 1, which proves that the multilayer structure of the graphene. The D peak is a low-strength peak and the 2D peak at ~1620 cm^−1^ is extremely weak (I_D_/I_G_ = 0.64), indicating that graphene only has few micro-defects.

### 3.2. Mechanical Testing of the PGSPS

**Figure 4:**
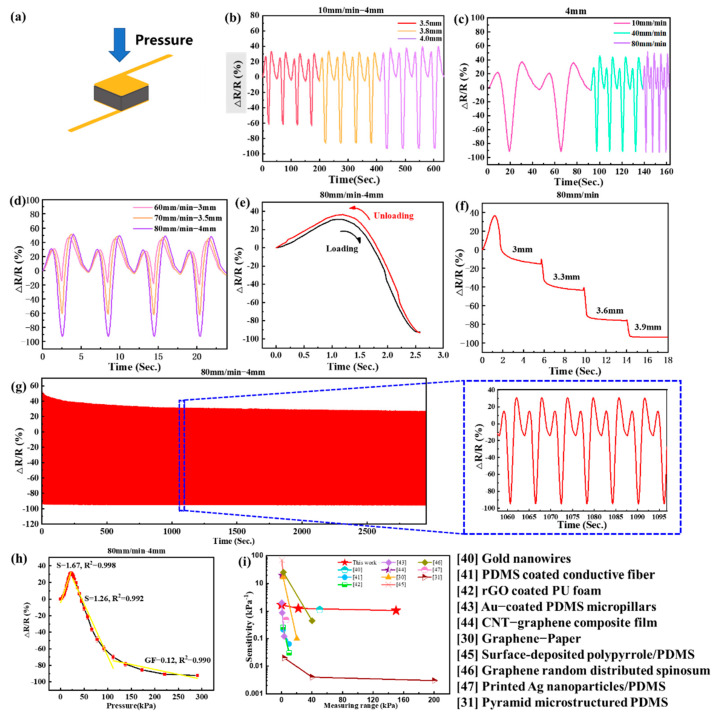
Single PGSPS performance test. (**a**) Schematic illustration of a PGSPS under pressure. (**b**) Different compression ranges test at the same speed. (**c**) Same compression range test at different speeds. (**d**) Different compression ranges test at different speeds. (**e**) Recovery curve of the PGSPS under exact range and speed. (**f**) Step compression test under exact speed. (**g**) Three thousand cycle tests under exact range and speed, five of these cycles are zoomed in on the right. (**h**) Gauge factor of the PGSPS in the working range. (**i**) Comparison of sensitivity–pressure characteristics with other flexible pressure sensors.

A variety of mechanical tests were conducted on a single PGSPS, for pressure sensing applications, in the form shown in Figure 4a. Figure 4b–h shows the change curves of the relative resistance rate, ΔR/R (%)-Pressure (kPa), of the PGSPS. The sensitivity of the PGSPS is generally defined as S = δ(ΔR/R_0_)/δP, where ΔR is the change in relative resistance, R_0_ is the initial resistance of the sensor when no pressure is applied, and P is the applied pressure value. Considering the thickness of the commonly used insole, PGSPSs with 5 mm thickness were tested. The ground reaction force at first touch is generally equal to the combination of body weight and acceleration, which is usually 120–140% of body weight at a normal pace [38]. Therefore, considering the ground reaction force, the regional plantar pressure is less than 150 kPa for the normal population [39]. This is the main reason why the PGSPS pressure measurement range is 0–250 kPa, but the maximum measured pressure in gait monitoring applications is 150 kPa. Besides, from Figure 4 we know that sensing performance performs well in this range. Under the 0–150 kPa insole sensing range, the final test compression speed is 80 mm/min, and the compression range is 4 mm. To verify the sensor performance under different pressures and compression ranges, several speeds close to 80 mm/min and several different compression ranges of about 4 mm were chosen to make sure that the PGSPS is adaptable to different situations. It can be seen from Figure 4b–d that no matter whether under same the same speed with different ranges, different speeds with the same range, or different speeds with different ranges, the shapes of the strain curves are almost identical. Moreover, different response strengths can also be distinguished. Figure 4e shows the resistance change in a typical loading and unloading circle with a velocity of 80 mm/min and a compression range of 4 mm, which shows the good recovery performance of the PGSPS. From the loop we can also see that the response time of the PGSPS is 120 ms and the recovery time is 50 ms (by applying pressure to compress the PGSPS to a thickness of 1 mm in the z-direction at 80 mm/min, the PGSPS reaches resistance stability again in 120 ms, and after the pressure was stopped, the device resumed its initial state in 50 ms), which is mainly derived from the elastic modulus and hole density of SBR foam, that is, in the early preparation of SBR foam type, the low elastic modulus of SBR foam leads to a long response recovery time. The step compression character of the sensor is shown in Figure 4f: At first the resistance increases when the pressure is low, which causes the horizontal spacing of conductive paths due to an increase in the lateral enlargement of the PGSPS. As the PGSPS is further compressed, the effect of the vertical density of the conductive network increasing exceeds the spacing increasing in the horizontal direction. Hence, the resistance of the PGSPS shows a uniform step change with the gradient compression. More specific trends can be seen in Figure 4h: the resistance of the PGSPS increases slightly under the low pressure, with a sensitivity of about 1.67 kPa^−1^, but decreases when the pressure exceeds 22 kPa, with the sensitivity decreasing. This is because under low pressure, the increasing of the horizontal spacing of conductive paths exceeds the effect that the vertical conductive path density increases. Moreover, the graphene sheets attached to the three-dimensional skeleton structure produce cracks under the action of low pressure, leading to an increase in resistance. Subsequently, as the pressure gradually increases, the increase in the vertical conductive path density exceeds the effect that the horizontal spacing of conductive paths increases, which causes the resistance to decrease rapidly and reach saturation after 150 kPa (After 150 kPa, the sensitivity gradually decreases to less than 0.12 kPa^−1^). The sensitivity of the PGSPS is reduced to its lowest (1.05 kPa^−1^) at the maximum operating pressure of 150 kPa. Because the gait monitoring application is designed to be used in the range of 0–150 kPa, we take the lowest sensitivity within this range as the device sensitivity. The linearity of the fitting curve (R^2^) is 0.990–0.998, which reflects the accuracy of calculating sensitivity. To verify the stability of the pressure sensor, cyclic tests of the PGSPS for in-shoe application conditions were conducted (Figure 4g). During the initial period of the 3000 cycle, the sensor’s resistance changes slightly lower, which comes from the stress release of the SBR foam and the shedding of graphene that is not tightly bound. The relative resistance change in subsequent loading–unloading loops almost remains the same, which shows good response and repetition characteristics of the device. The change in the relative resistance rate with pressure is consistent with the sensitivity curve in Figure 4h, which proves that the sensor has good stability and a long service life.

Compared with existing flexible pressure sensors [30,31,40,41,42,43,44,45,46,47], our PGSPS presents distinguished superiority in the pressure detection range and sensitivity (Figure 4i). With a wider pressure range than most wearable pressure sensors [30,40,41,42,43,44,45,46,47], the PGSPS is particularly suitable for gait monitoring applications, which requires large pressure ranges. Besides, when compared with a large-range pressure sensor [31], the sensitivity of the PGSPS was significantly higher in its application range of 0–150 kPa.

To illustrate high linearity, a fast response speed, and high stability of the PGSPS, while realizing comparisons between devices with different sensitivities and pressure ranges, we utilized the linearity range to measure device linearity, using the ratio of the test pressure and its corresponding response time to measure the response speed, using the maximum test pressure and the corresponding cycles to measure stability. As shown in Table 2, the PGSPS has a two-stage wide linearity range and can achieve a greater response per unit time. Although the number of cycles was not the highest, they were completed at the largest range of 150 kPa.

These results show its application potential in the field of flexible gait monitoring. Besides, in terms of the potential for large-scale application, by combining the performances with the low-cost process mentioned above, the PGSPS take cost and performance into consideration, which helps to standardize the process for mass production.

### 3.3. Construction and Testing of the Preliminary PGSPS Insole System

**Figure 5:**
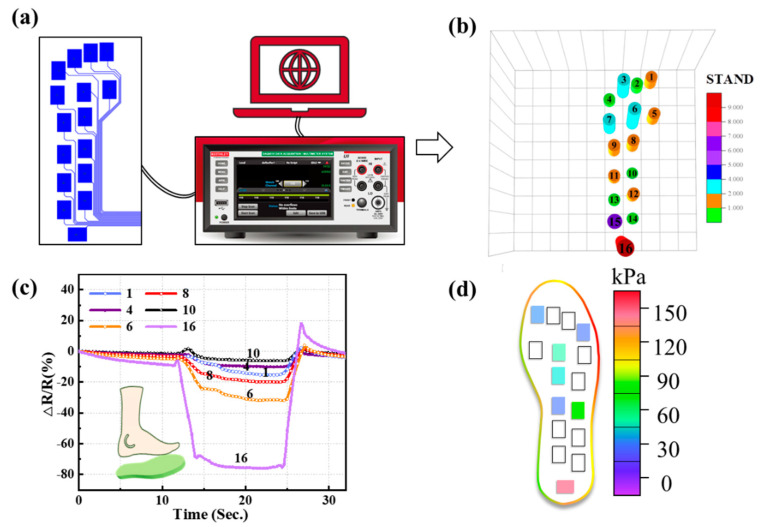
(**a**) Diagram of gait testing using commercial instruments. (**b**) Pressure nephogram of the in-shoe system with an adult standing on it. (**c**) Test results of six sensing points (1, 4, 6, 8, 10, and 16) at static stations. (**d**) Pressure map reconstructed from (**c**).

After sixteen identical PGSPSs are integrated into the insole and connected with the FPC processing module, the preliminary insole sensor system is obtained. Table 3 shows the characters of the insole and the subject condition. To achieve high-precision testing, a 7^1/2^ digital multimeter was used to monitor sixteen sensors at the same time, and the waveforms are displayed on the screen in real-time. After taking a negative of the resistance change, normalizing it, and multiplying it by ten, a pressure nephogram was drawn. As shown in Figure 5b, when an adult was standing on the insole, sixteen PGSPSs measured and displayed the pressure distribution, which corresponds to the standing posture of the person. Next, the dynamic waveform of a step is monitored and displayed; waveforms of six typical sensing points (1, 4, 6, 8, 10, and 16) were chosen and shown in Figure 5c. The PGSPS in the corresponding position records the whole process of a step, which matches the waveform of the individual device. Meanwhile, the different pressure distribution is displayed by the amplitude of the waveform (Figure 5d); the highest plantar pressure of the subject was 139 kPa, within the designed gait monitoring range of the PGSPS. The insole test shows the uniformity and stability of the PGSPS, and further confirms the usability of the insole sensor system. Furthermore, we will learn the performance changes of the PGSPS insole in a shoe, conduct system integration design, and realize real-time gait monitoring and wireless transmission in the next section.

### 3.4. Construction and Testing of the Low-Cost, Portable, and Wireless In-Shoe System

In the previous section, a preliminary PGSPS insole sensor system was finished and the pressure distribution of an adult standing and stepping was measured. However, for a daily use gait monitoring system, it should have good performance in shoes, and the whole system should be customizable, portable, and wireless. Therefore, in this part, the in-shoe and on-floor performance of the system are shown, and a portable and wireless processing module is designed and tested. Finally, a completely flexible in-shoe system based on the PGSPS will be shown, and a customizable, low-cost design will be discussed.

**Figure 6:**
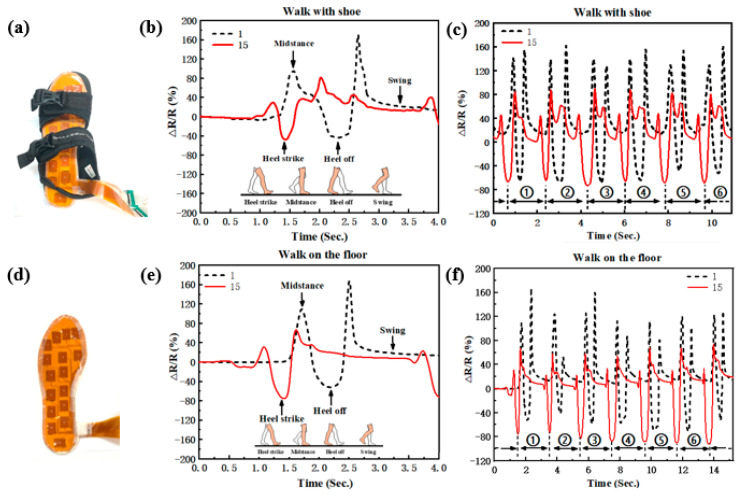
Gait cycle test using a commercial instrument, DM6510. (**a**) Diagram of walking with a shoe. (**b**) Resistance response of sensor points 1 and 15 when walking in shoes. (**d**) Diagram of walking on the floor. (**e**) Resistance response of sensor points 1 and 15 when walking on the floor. (**c**,**f**) Gait cycle test while walking in shoes and walking on the floor, respectively.

To verify the performance changes between in-shoe and on-floor usage, a walking test was carried out. As shown in Figure 6, insole systems in sandals (foot movements can be seen) and on the floor are tested in the same way. To see the gait pressure changes, the first PGSPS (hallux) in region one and the fifteenth PGSPS (heel) in region four were chosen to be displayed. The subject was asked to walk at the same pace, and the system in-shoe or on-floor (out-shoe) recorded the gait information, then took the information about the first six steps (Figure 6c,f) and amplified the first step (Figure 6b,e). In both cases, the system recorded complete gait information, including the four stages of walking (heel strike, midstance, heel-off, and swing). The two cases exhibit approximate waveforms, which confirms the flexible insole system can be used in-shoe. However, compared with walking on the floor, the waveform of walking with shoes shows more volatility, which is mainly caused by two factors: (i) The insole system was not fixed to the sole during use and moves relative to the sole when walking, which is especially noticeable for sandals. (ii) Compared with the floor, the sole of the shoe is not flat, which brings an initial deformation to the insole system. All in all, the current insole system can monitor gait in shoes clearly, and the problem can be solved by customizing the design, improving the insole surface friction, or using non-destructive fixation methods.

**Figure 7:**
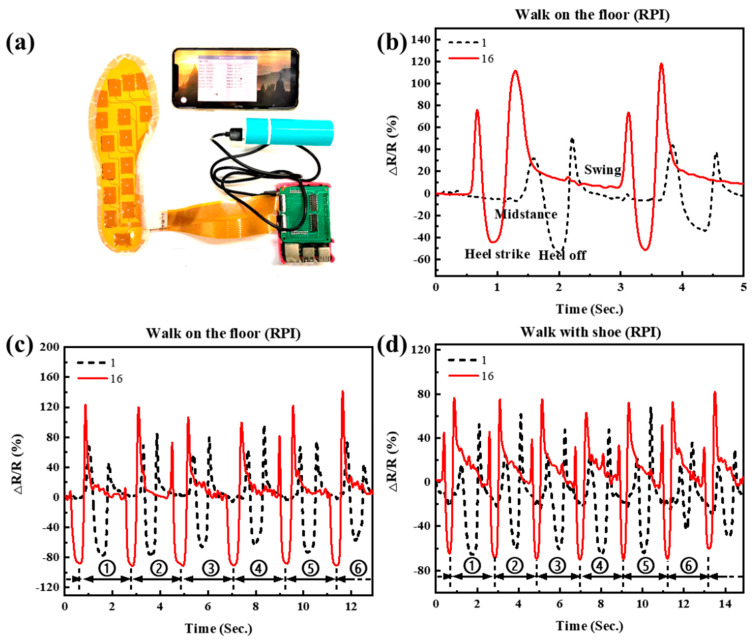
(**a**) Diagram of gait cycle test using RPI system. (**b**) Resistance response of sensor points 1 and 16 when walking on the floor. (**c**,**d**) Gait cycle test while walking in shoes and walking on the floor, respectively.

Furthermore, in order to realize a portable and wireless insole system, a system consisting of a PGSPS-based insole sensor system with a double-faced insole-shaped flexible printed circuit board (FPC), a raspberry PI system (RPI), a data acquisition (DAQ) circuit, and data processing and displaying software (on a smartphone) was designed and fabricated. As shown in Figure 7a, the sensor part (insole) was connected to the processing unit through the FPC. The RPI and DAQ circuits were integrated with Bluetooth and battery. The gait information was finally transmitted to a smartphone through Bluetooth and displayed by the software. As depicted in Figure 7b–d, in contrast to the former part, this time the first PGSPS (hallux) in region one and the sixteenth PGSPS (heel) in region four were chosen to be shown. The results indicate that the amplitude of the 16th PGSPS’s waveform is significantly larger than that of the 15th PGSPS’s, noted above, due to the 16th PGSPS being located at the back of the heel, which undertakes more pressure than the 15th PGSPS, and the same conclusion can be drawn from the pressure nephogram in Figure 5b. Moreover, no matter whether walking in-shoe or on-floor, the RPI system can clearly record the gait information in real-time. Next, a comparison between the measurement results of the portable system and a high-precision digital multimeter will be shown.

### 3.5. Comparison between the Portable Insole System and a High-Precision Multimeter, and Tests of Several Different Gaits

**Figure 8:**
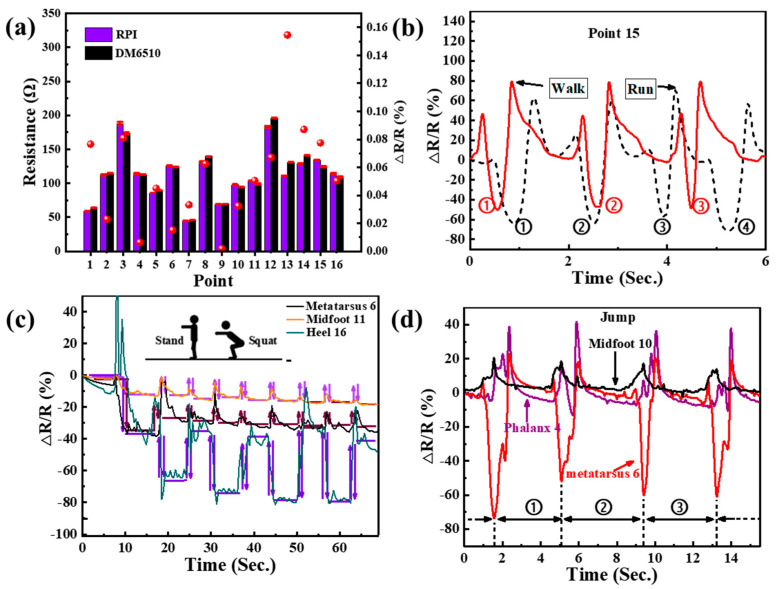
(**a**) Comparison of the resistance results of all 16 PGSPSs using the RPI system and DM6510, respectively, and the measurement error. (**b**) A gait test comparison between running and walking from sensor point 15. (**c**) Resistance response of sensors 6, 11, and 16 during squatting. (**d**) Resistance response of 4 (phalanx), 6 (metatarsus), and 10 (midfoot) during jumping.

To verify that the performance of the portable insole system is suitable, a waveform comparison between the RPI system and a DM6510 high-precision digital multimeter was carried out. The sensing results of all sixteen PGSPSs using the RPI system and DM6510 as well as the measurement error are shown in Figure 8a. For the same test conditions, the resistance changes measured by the two methods are roughly the same, but the error is a bit large at the 13th point. As shown in Figure 2b, the 13th point is at the back of the midfoot region, on the inside of the foot; this position does not fit well with the sole [48], which causes a slightly larger error. The opposite situation occurs in the 9th point, located on the forepart of the midfoot (near the metatarsus), which always fits well with the sole during walking [49]. The error can be reduced by customization. On the other hand, the 13th point usually reflects a normal gait with a small or no obvious change in pressure.

In addition to the gait monitoring, the portable insole system can also monitor running, standing, squatting, and jumping. Figure 8b shows waveforms between walking and running, and the 15th point, with an obvious change, was selected for analysis. Except for a higher frequency, the waveform amplitude of running is significantly different from that of walking; in particular, the heel strike process is more severe while the heel-off process is more moderate. This is because the acceleration is greater in the heel strike process of the running state, resulting in a greater ground reaction force [50]. The heel-off process is shortened [51], which results in a shorter recovery time.

Figure 8c shows the monitoring of squatting. The waveforms of point six in the metatarsus, eleven in the midfoot, and sixteen in the heel were chosen to show the movements. During the crouching and getting up, the waveforms change rapidly and maintain a fixed value when the body reaches a stable state. Besides, the heel is under the highest pressure, which corresponds to the actual situation.

For jumps, the signal is more intense. Points four (phalanx), six (metatarsus), and ten (midfoot) are suitable for jump monitoring and were selected for analysis [52]. Compared with walking, the subject suffered a greater impact on the metatarsus, and the waveforms show more vibration, which comes from the body vibration when jumping. Besides, the phalanx and metatarsus region have more vibration during the heel-off process, which comes from the separation between the insole and the middle/back of the sole when jumping up.

In summary, it has been verified that a flexible, portable, and wireless in-shoe system based on the PGSPS can satisfy monitoring multiple gaits in daily life and reflect the characteristics of the corresponding gait clearly. Besides, the system integrates the sensing module, the signal processing unit, the wireless transmission module, the display software, and the data processing module. The miniaturized system can be worn with shoes and paired with a smartphone for daily gait monitoring. The SBR foam and graphene nanoplate paste used in this work are low cost, and the uniform and stable performance of the PGSPS is suitable for large-scale production. Moreover, a raspberry PI can be changed into a dedicated signal processing circuit, which will greatly reduce the cost and size of the processing unit, making it suitable for in-shoe applications. In addition to the current IOS app, software suitable for other systems can be designed so that users can conduct daily gait monitoring on their smartphones. The system has potential for guiding gait training and detecting diseases, and can be used for the daily tracking of motion as well as rehabilitation training. However, in the following work, it is also necessary to solve various problems: (i) To further improve the signal quality. The contact problem between the PGSPS insole and the sole should be solved, and the PGSPS structure should be iteratively optimized. (ii) To improve the ability of the system by adding gait analysis for non-healthy states, and increasing the system’s monitoring stability in running and jumping. (iii) A better man–machine interaction can be achieved by analyzing the impact of customization design on performance, and giving the software abnormal gait judgment ability through the application of intelligent algorithms, which will help to realize a smart gait monitoring system.

To optimize our device and system according to actual requirements, we are currently working with a hospital to find solutions for the diagnosis of cerebral palsy through gait monitoring. As a neurological disease, cerebral palsy can be directly reflected in the patient’s gait [3]. Traditional gait detectors, including the step table gait analyzer, are expensive and immobile, which are not suitable for long-term monitoring and diagnosis. Our low-cost and portable system can meet these needs, in line with the needs of this disease diagnosis.

## 4. Conclusions

In this work, a low-cost, portable, and wireless in-shoe system based on flexible porous graphene–SBR foam was designed and fabricated. A PGSPS with a biomimetic structure based on vertebrates’ skeleton and attached tissues was designed, which realized a large-range and high-sensitivity porous graphene–SBR pressure sensor for gait monitoring that can be integrated into shoes. The system has the complete ability to collect, transmit, process, and display gait information, which can realize real-time monitoring of a variety of peoples’ gait through a smartphone. Compared to a high-precision digital multimeter, it is more portable, suitable for daily use, and the accuracy of the waveform meets the judgment requirements. The system has potential in monitoring the safety of the elderly, which is very helpful in today’s society with an increasingly aging population. It can also guide gait training and detect diseases, as well as be used for the daily tracking of motion and rehabilitation training. In follow-up work, an intelligent diagnosis algorithm can be developed to realize a smart gait monitoring system.

## Figures and Tables

**Table 1:** The principal characteristics of the SBR foam used in PGSPS.

Parameter	Units	Value
Tensile strength	MPa	18
Density	g/cm^3^	0.12
Hole size	μm	20–250
Length	mm	15
Width	mm	10
Height	mm	5

**Table 2:** Comparison of characteristics with other flexible pressure sensors.

Reference	Linearity Range (kPa)	Response Speed (kPa/ms)	Stability (Maximum Pressure Cycles)
This work	0–22, 22–150	1.25	150 kPa–3000
[35]	0–1, 1.2–1.8	0.01	None ^1^
[40]	0–50	0.15	2.5 kPa–50,000
[43]	0–0.22, 0.22–1, and 1–3.5	4.00 × 10^−4^	0.02 kPa–10,000
[44]	0–0.27	3.59 × 10^−5^	0.15 kPa–35,000
[46]	0–0.5, 0.5–2	3.33 × 10^−4^	0.25 kPa–1000
[47]	0–2.6, 2.6–40	8.33 × 10^−3^	1.5 kPa–3000

^1^ This capability is not mentioned in the article.

**Table 3:** The characters of the insole and the subject condition.

Insole Size (EU Size)	Thickness (mm)	Materials (Apart from PGSPS)	Weight of the Subject (kg)	Height of the Subject (cm)
37	5	SBR foam	60	163

## Data Availability

The data presented in this study are available on request from corresponding author.
